# Polyamine Analog Diethylnorspermidine Restricts Coxsackievirus B3 and Is Overcome by 2A Protease Mutation In Vitro

**DOI:** 10.3390/v13020310

**Published:** 2021-02-16

**Authors:** Bridget M. Hulsebosch, Bryan C. Mounce

**Affiliations:** 1 Department of Microbiology and Immunology, Stritch School of Medicine, Loyola University Chicago, Maywood, IL 60153, USA; bhulsebosch@luc.edu; 2 Infectious Disease and Immunology Research Institute, Stritch School of Medicine, Loyola University Chicago, Maywood, IL 60153, USA

**Keywords:** Coxsackievirus, polyamines, enterovirus, protease

## Abstract

Enteroviruses, including Coxsackievirus B3 (CVB3), are pervasive pathogens that cause significant disease, including cardiomyopathies. Unfortunately, no treatments or vaccines are available for infected individuals. We identified the host polyamine pathway as a potential drug target, as inhibiting polyamine biosynthesis significantly reduces enterovirus replication in vitro and in vivo. Here, we show that CVB3 is sensitive to polyamine depletion through the polyamine analog diethylnorspermidine (DENSpm), which enhances polyamine catabolism through induction of polyamine acetylation. We demonstrate that CVB3 acquires resistance to DENSpm via mutation of the 2A protease, which enhances proteolytic activity in the presence of DENSpm. Resistance to DENSpm occurred via mutation of a non-catalytic site mutation and results in decreased fitness. These data demonstrate that potential for targeting polyamine catabolism as an antiviral target as well as highlight a potential mechanism of resistance.

## 1. Introduction

Enterovirus infections are one of the most abundant viral infections in the United States and cause a wide range of diseases, ranging from mild to severe [1,2,3]. Coxsackievirus B3 (CVB3) is a positive-sense, non-enveloped enterovirus, and like all enteroviruses, belongs to the *Picornaviridae* family [4]. CVB3 infection has a wide range of symptoms, including fever, dilated cardiomyopathy, aseptic meningitis, and is the leading cause of viral myocarditis. Importantly, CVB3 persists in cardiac tissue [1,3,4,5,6]. There is no vaccine or antivirals currently available for this virus, emphasizing the importance of identifying novel antiviral targets to control infection and spread.

**Figure 1:**
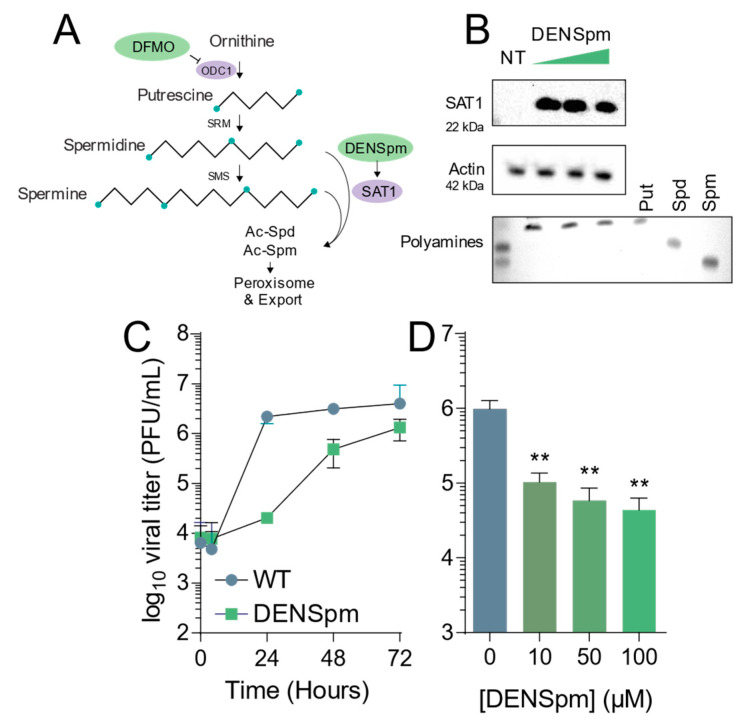
DENSpm induces polyamine depletion and limits Coxsackievirus B3 infection. (**A**) Schematic of the mammalian polyamine biosynthetic pathway. Pertinent inhibitors are highlighted in green, while important enzymes are highlighted in lilac. (**B**) Vero-E6 cells were treated with 10, 50, and 100 μM DENSpm for 16 h prior to collection for Western blot for SAT1, above, and thin layer chromatography for polyamines measuring the presence of putrescine (Put), spermidine (Spm), and spermine (Spm), below. (**C**) Cells were left untreated (NT) or treated with 100 μM DENSpm for 16h prior to infection at multiplicity of infection of 0.01 CVB3. Viral titers were determined by plaque assay at the times indicated. (**D**) Cells were treated with escalating doses of DENSpm for 16h prior to infection at MOI 0.01. Viral titers were determined at 48 hpi. ** *p* ≤ 0.01using Student’s *t*-test (*n* ≥ 3), comparing treated samples to untreated controls. Error bars represent ± 1 SEM.

Polyamines are small, flexible, positively charged carbon chains crucial for mammalian cell function [7,8,9]. They have been known to be crucial for DNA and RNA structure, protein–RNA interactions, and translation and are found in all cell types [7,8]. Their biosynthesis pathway begins with the polyamine precursor molecule, ornithine, which is converted to the first polyamine, putrescine, via the rate-limiting enzyme ornithine decarboxylase 1 (ODC1) [7,8]. Putrescine is then further converted to spermidine and spermine (Figure 1A), which can both then be catabolized back to putrescine or marked for degradation and export via spermidine/spermine acetyltransferase 1 (SAT1) [7,8]. These two enzymes, ODC1 and SAT1, are critical in the synthesis and degradation of polyamines.

Polyamines are essential for some viruses and their successful replication as well and have even been shown to be incorporated into the virions by some DNA and RNA viruses [9,10,11,12]. They are necessary for many stages in viral replication cycles, and the polyamine biosynthesis pathway has been effectively targeted for parasite and cancer treatments [13,14,15]. Recent studies showed that targeting polyamine metabolism with compounds like difluoromethylornithine (DFMO), a suicide inhibitor of ODC1 that effectively inhibits polyamine synthesis [15,16], is an effective antiviral therapy in vitro and in vivo [10,17,18]. The compound diethylnorspermidine (DENSpm) is a polyamine analog that activates SAT1, promoting the catabolism of spermidine and spermine back to putrescine or leading to their degradation in peroxisomes [14,19]. Specifically, polyamines facilitate CVB3 infection and depleting cells of polyamines with DFMO inhibits CVB3 replication [18]; however, the virus is able to overcome this depletion through enhanced 2A and 3C protease activity [12,18]. It is unknown if CVB3 is able overcome this depletion from another polyamine-depleting compound besides DFMO.

Recent studies have shown the inhibition or depletion of cellular polyamines restricts virus replication and that polyamines play important roles in virus replication [12,17,18,20]. Polyamines enhance or stimulate the activity of cellular proteins [7,8] and are also critical for some DNA viral proteins, including a viral kinase of Varicella-Zoster virus and a viral polymerase of vaccinia virus [21,22]. In a study by Dial et al., both the 2A and 3C viral proteases of CVB3 rely on polyamines for their proteolytic functions, and depleting cellular polyamines inhibits the catalytic activity of the proteases and replication [18]. However, the relationship between viral proteases and polyamines has yet to be completely characterized.

Viral proteases have diverse roles during infection, including enterovirus proteases. CVB3 encodes two distinct proteases, 2A and 3C. These two proteases catalyze the majority of cleavage of the viral polyprotein [23,24,25]. Upon entry into the cell, CVB3’s RNA genome is translated by the host cell’s machinery, synthesizing the polyprotein [23,24,25]. 2A performs the primary cleavage of the polyprotein, and 3C catalyzes subsequent polyprotein cleavage [23,25]. This 2A protease also has many cellular targets, most notably eIF4G, an important cap-dependent translation factor, and its cleavage shuts off host protein translation, thereby enhancing IRES-mediated CVB3 translation [23,25,26]. Due to the importance and necessity of both the 2A and 3C proteases for CVB3 infection, protease inhibitors are an effective antiviral target in CVB3 infection [27,28].

To explore the relationship between polyamines and viruses, specifically their proteases, we screened for an escape mutant of CVB3 from polyamine depletion via DENSpm treatment. By passaging CVB3 in DENSpm-treated cells, we identified a 2A protease mutant, 2A^S35G^. We cloned this mutant into the CVB3 genome and observed its stability and fitness compared to wildtype virus. We determined the mutant’s sensitivity to both DENSpm and DFMO treatment and examined if this mutant exhibits 2A-mediated proteolytic activity in the absence of polyamines using protease-dependent split-luciferase reporter system, as well as the mutant’s ability to cleave cellular targets. These data further contribute to our understanding of polyamines in viral infections and the evolution of viruses while under pressure from potential antiviral therapies.

## 2. Materials and Methods

### 2.1. Cell Culture

Cells were maintained at 37 °C in 5% CO_2_, in Dulbecco’s modified Eagle’s medium (DMEM; Life Technologies, Waltham, MA, USA) with bovine serum and penicillin–streptomycin. Vero (BEI Resources) and HeLa cells were supplemented with 10% new-born calf serum (NBCS; Thermo-Fischer, Waltham, MA, USA). 293T and Huh7 cells, kindly provided by Susan Uprichard, were supplemented with 10% fetal bovine serum (FBS; Thermo-Fischer).

### 2.2. Generation of ^2AS35^ Mutants

CVB3 (Nancy strain) [29] was derived from the first passage of the virus in Vero cells after rescue from the infectious clone. Briefly, the CVB3 infectious clone [30] was linearized with SapI (New England Biolabs (NEB)) and used to generate RNA in vitro. This RNA was transfected into Vero cells to recover virus. The 2A^S35G^ mutant viruses were generated via site-directed mutagenesis of the wildtype plasmid using primers 5′-C TGG CAA AAC TGT GTG TGG GAA GGT TAC AAC AGA GAC CTC-3′ (forward) and 5′-GAG GTC TCT GTT GTA ACC TTC CCA CAC ACA GTT TTG CCA G-3′ (reverse).

### 2.3. Infection and Enumeration of Viral Titers

CVB3 was derived from the first passage of virus in Vero cells, which were obtained through BEI Resources, National Institutes of Allergy and Infectious Diseases, National Institutes of Health (NR-10385). For all infections, DENSpm and DFMO were maintained throughout infection as designated. Viral stocks were maintained at −80 °C. For infection, virus was diluted in serum-free DMEM for a multiplicity of infection (MOI) of 0.1 on Vero cells, unless otherwise indicated. The viral inoculum was overlain on cells for 10 to 30 min, and the cells were washed with PBS before replenishment of media. Supernatants were collected from CVB3 24 hpi and 48 hpi, or as indicated. Dilutions of supernatant were prepared in serum-free DMEM and used to inoculate confluent monolayers of Vero cells for 10 to 15 min at 37 °C. Cells were overlaid with 0.8% agarose in DMEM containing 2% NBCS. Samples were incubated for 2 days at 37 °C. Following incubation, cells were fixed with 4% formalin and revealed with crystal violet solution (10% crystal violet; Sigma-Aldrich, St. Louis, MO, USA). Plaques were enumerated and used to back-calculate the number of plaque-forming units (pfu) per milliliter of collected volume.

### 2.4. CVB3 Serial Passage

Vero cells were treated with DENSpm 16 h before infection with CVB3 at MOI of 0.1. After 24 h, 1/10th of cell culture volume was used to inoculate the next passage. This process was continued for ten passages, at which time viral RNA was purified from the cellular supernatant, reverse transcribed, amplified using CVB3 specific primers [18], and Sanger sequenced. Sequences were aligned to CVB3 parental genome, and mutants were confirmed by manual chromatogram inspection.

### 2.5. Drug Treatments

N1,N11-Diethylnorspermine (DENSpm; Santa Cruz Biotechnology, Santa Cruz, CA, USA) and Difluoromethylornithine (DFMO; TargetMol, Boston, MA, USA) were diluted to 100× solution (10 mM and 100 mM, respectively) in sterile water. For DENSpm treatment, cells were trypsinized and reseeded with fresh medium supplemented with 2% serum. Following overnight attachment, cells were treated with DENSpm as indicated for 16 h to allow for complete depletion of polyamines. For DFMO treatment, cells were treated 96 h prior to infection. During infection, media were cleared and saved from cells. The same media containing DENSpm and DFMO were then used to replenish the cells following infection. Cells were incubated at the appropriate temperature for the duration of infection.

### 2.6. RNA Purification and cDNA Synthesis

Media were cleared from cells, and Trizol reagent (Zymo Research, Irvine, CA, USA) was directly added to cells or supernatant. The lysate was then collected, and RNA was purified according to the manufacturer’s protocol utilizing the Direct-zol RNA Miniprep Plus Kit (Zymo Research, Irvine, CA, USA). Purified RNA was subsequently used for cDNA synthesis using High-Capacity cDNA Reverse Transcription Kits (Thermo-Fischer), according to the manufacturer’s protocol, with 10–100 ng of RNA and random hexamer primers.

### 2.7. DENSpm and DFMO Sensitivity Assays

Vero cells were treated with either 500 nM to 100 μM DENSpm for 16 h or 100 μM to 1mM DFMO for 4 days prior to infection with CVB3 at an MOI of 0.1. At 24 hpi, supernatant was collected and titers were determined. Titers measured after drug treatment were divided by titers without treatment to obtain percent replication compared to control untreated conditions.

### 2.8. Stability and Competition Assays

To measure the stability of the mutations, Vero cells were infected at an MOI of 0.1 with the viral mutant for 24 h. The virus was then passed to new cells by transferring 50 μL supernatant. After five passages, RNA was extracted and purified from supernatants and reverse transcribed. Sanger sequencing was used to determine whether mutations were stable over the passages by looking at the chromatograms and determining the presence or absence of the mutant nucleotide as previously described [18]. Competition assays were similarly performed, but Vero cells were infected at an MOI of 0.1 with an equal combination of wildtype and mutant CVB3 and passaged five times. Fitness was determined via Sanger sequencing and analysis of the chromatogram to determine if the wildtype or mutant nucleotide was present in the sample.

### 2.9. Protease Plasmid Cloning

Primers were designed to target the wildtype 2A protease as previously described [18]. CVB3 plasmids containing the 2A protease were used to clone the mutant protease. To target the 2A and 2A mutant protease, the primers included NotI and XbaI recognition sites. Protease sequences were amplified via PCR and cloned into the pFLAG-CMV vector. Clones were verified for sequencing (GenScript, Piscataway, NJ, USA). Oligonucleotide sequences corresponding to the amino acid sequence for the wildtype and mutant 2A and 3C protease targets were designed and cloned into the pGlo-3F vector and verified by sequencing (GenScript) as previously described [18].

### 2.10. Transfections

293T or Vero cells were plated at 80%–90% confluency and either treated with 10 μM, 50 μM, or 100 μM DENSpm for 16 h or left untreated. The plasmids were then transfected in the combinations described in the figures, according to the manufacturer’s protocol, using LipoD293 (SignaGen Laboratories). The transfection was incubated at 37 °C for 24 h.

### 2.11. Luciferase Protease Assay

Veros were treated with 10 μM, 50 μM, or 100 μM for 16 h or left untreated. They were then transfected using LipoD293 (SignaGen Laboratories, Rockville, MD, USA) with the 2A substrate alone, 2A substrate plus the 2A WT protease or 2A mutant protease, and a TK Renilla transfection control plasmid. For luciferase assays, cells were combined with firefly substrate followed by subsequent Renilla (Stop and Glo; Promega, Madison, WI, USA) luciferase substrate 24 h post-transfection. Luciferase assays were performed according to the manufacturer’s recommendations (Promega), and results were measured via the Veritas Microplate Luminometer (Turner BioSystems, Promega). Protease activity was determined by diving firefly luciferase activity by the Renilla luciferase activity control and normalizing to untreated samples.

### 2.12. Western Blots

Samples were collected with Bolt LDS Buffer and Bolt Reducing Agent (Invitrogen, Waltham, MA, USA) and run on polyacrylamide gels. Gels were transferred using the iBlot 2 Gel Transfer Device (Invitrogen). Membranes were probed with primary antibodies for eIF4G, (1:1000, Santa Cruz Biotechnology), actin (1:2000, ProteinTech, Rosemont, IL, USA), SAT1 (1:100, Santa Cruz Biotechnology), and GAPDH (1:1000, Santa Cruz Biotechnology). Membranes were treated with SuperSignal West Pico PLUS Chemiluminescent Substrate (ThermoFisher Scientific) and visualized on FluorChem E imager (Protein Simple, San Jose, CA, USA).

### 2.13. Plaque Size Measurement

Vero cells were seeded in 10 cm dishes and grown to confluence. Approximately 30 plaque-forming units (PFU) of each mutant was diluted in a 2.5 mL inoculum of serum-free DMEM. The media on the Vero cells were aspirated and replaced with the 2.5 mL inoculum containing the virus. The inoculum was incubated on the cells for approximately 30 min at 37 °C. After 30 min, an overlay of 8 mL 0.8% agarose was added to each dish. The dishes were incubated at 37 °C for 2 days to allow plaque formation. The cells were fixed with 4% formalin and the agarose plugs removed. The fixed cells were stained with crystal violet. Plaque size was determined using ImageJ software (Version 1.51k) [31].

### 2.14. Thin Layer Chromatography Determination of Polyamines

Polyamines were separated by thin-layer chromatography as previously described [32]. For all samples, cells were treated as described prior to being trypsinized and centrifuged. Pellets were washed with PBS and then resuspended in 200 μL 2% perchloric acid. Samples were then incubated overnight at 4 °C. Supernatant (200 μL) was combined with 200 μL 5 mg/mL dansyl chloride (Sigma Aldrich) in acetone and 100 μL saturated sodium bicarbonate. Samples were incubated in the dark overnight at room temperature. Excess dansyl chloride was cleared by incubating the reaction with 100 μL 150 mg/mL proline (Sigma Aldrich). Dansylated polyamines were extracted with 50 μL toluene (Sigma Aldrich) and centrifuged. Five microliters of sample was added in small spots to the TLC plate (silica gel matrix; Sigma Aldrich) and exposed to ascending chromatography with 1:1 cyclohexane/ethyl acetate. The plate was dried and visualized via exposure to UV.

### 2.15. Statistical Analysis

Prism 6 (GraphPad) was used to generate graphs and perform statistical analysis. For all analyses, two-tailed Student’s t-test was used to compare groups, unless otherwise noted, with a = 0.05. For tests of sample proportions, p values were derived from calculated Z scores with two tails and a = 0.05. Half-maximal inhibitory concentration (IC50) values were calculated using Prism 6 using the built-in analysis tool.

## 3. Results

### 3.1. DENSpm Induces Polyamine Depletion and Limits Coxsackievirus B3 INFECTION

DENSpm induces polyamine depletion via the upregulation of SAT1 and concomitant acetylation, interconversion, and degradation of the polyamines spermidine and spermine. To confirm that DENSpm induces these changes, we treated Vero-E6 cells with increasing doses of DENSpm and measured translation of SAT1 by Western blot. We observed increasing signal for SAT1 with increasing doses, from 10 μM to 100 μM (Figure 1B, above). Additionally, we measured cellular polyamines by thin layer chromatography. As expected, we found that DENSpm treatment correlated with depletion of spermidine and spermine and an increase in putrescine (Figure 1B, lower). To determine if this polyamine depletion affected viral infection, we infected cells treated with 100 μM DENSpm with CVB3 at a multiplicity of infection (MOI) of 0.01 and measured viral titers over 72 h. We observed that viral titers were significantly reduced throughout infection, though viral titers nearly reached untreated levels by 72 h (Figure 1C). Finally, we infected cells treated with increasing concentrations of DENSpm and infected with CVB3 at MOI 0.01, measuring viral titers at 48 h. We observed that viral titers decreased (Figure 1D), suggesting that DENSpm restricts virus replication both in a dose-dependent manner and over several rounds of replication.

### 3.2. Coxsackievirus B3 Gains Resistance to DENSpm via 2A Mutation in 2A Protease

**Figure 2:**
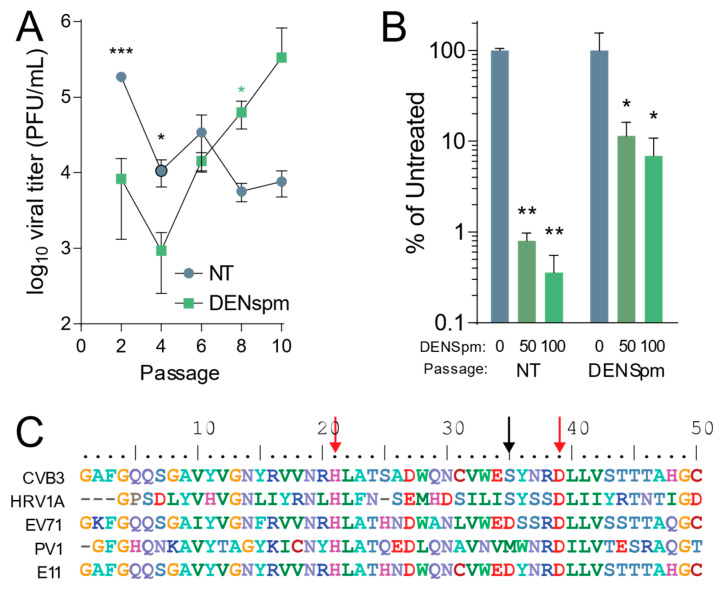
Coxsackievirus B3 gains resistance to DENSpm via mutation in 2A protease. (**A**) Vero-E6 cells were left untreated or treated with 100 μM DENSpm for 16 h prior to infection with CVB3 at an MOI 0.1. Virus was collected at 24 hpi and used to inoculate the next passage. Viral titers were determined via plaque assay for the passages shown. (**B**) CVB3 passaged 10 times over Vero-E6 cells, either treated with 100 μM or untreated, were used to infect Vero cells treated with increasing doses of DENspm for 24 hpi. Viral titers were determined by plaque assay. (**C**) Partial 2A protease sequence of CVB3 compared to other enteroviruses. Red arrows indicate amino acid residues of the protease active site, and the black arrow indicates mutated amino acid residue of 2A^S35^ mutants. * *p* ≤ 0.05, ** *p* ≤ 0.01, *** *p* ≤ 0.001 using Student’s *t*-test (*n* ≥ 3), comparing treated samples to untreated controls. Error bars represent ± 1 SEM.

After establishing that CVB3 is sensitive to depletion of cellular polyamines by DENSpm, we wished to determine the evolution of CVB3 after multiple replication cycles in cells depleted of cellular polyamines after DENSpm treatment. Our previous work showed that CVB3 gains resistance to cellular polyamine depletion from DFMO treatment through both a 2A protease mutation and a 3C protease mutation [18]. To determine if the same or different mutations emerged with passage in DENSpm treatment, CVB3 was passaged ten times in Vero-E6 cells treated with 100 μM DENSpm 16 h pre-infection or left untreated. The cells were then infected with CVB3 at an MOI of 0.1. After 24 h post infection (hpi), 1/10 of the supernatant was passaged and used to inoculate the next set of cells. Viral titers were determined per passage and multiple passages (Figure 2A). After 10 passages, the virus that was passaged in cells treated with DENSpm exhibited higher titers compared to that of virus passaged in untreated cells. This difference in titers suggested that this virus from DENSpm-treated cells gained resistance to DENSpm. Supernatant from the 10th passage of virus passaged in DENSpm-treated cells and untreated cells was then used to measure resistance to DENSpm, infecting cells with increasing doses of the drug. We found that CVB3 that was passaged in cells treated with DENSpm did not appear to be sensitive to DENSpm treatment at 50 μM and 100 μM compared to virus that was repeatedly passaged in untreated cells, which had a significant decrease in titers (Figure 2B). We extracted viral RNA of the 10th passage, purified, reverse transcribed, and Sanger sequenced. When aligning with the parental genome, an S35G mutation was found in the 2A protease (black arrow) (Figure 2C). It is important to note that this S35G mutation does not occur in the active site (red arrows) of the 2A protease, and no mutations were found in virus that was passaged in untreated cells. While the serine at position 35 of the 2A protease is conserved between CVB3 and the distantly related human rhinovirus 1A (HRV1A), other enteroviruses exhibit distinct amino acids at this site, and it is not strictly conserved.

### 3.3. CVB3 2A^S35G^ Confers Resistance to DENSpm

**Figure 3:**
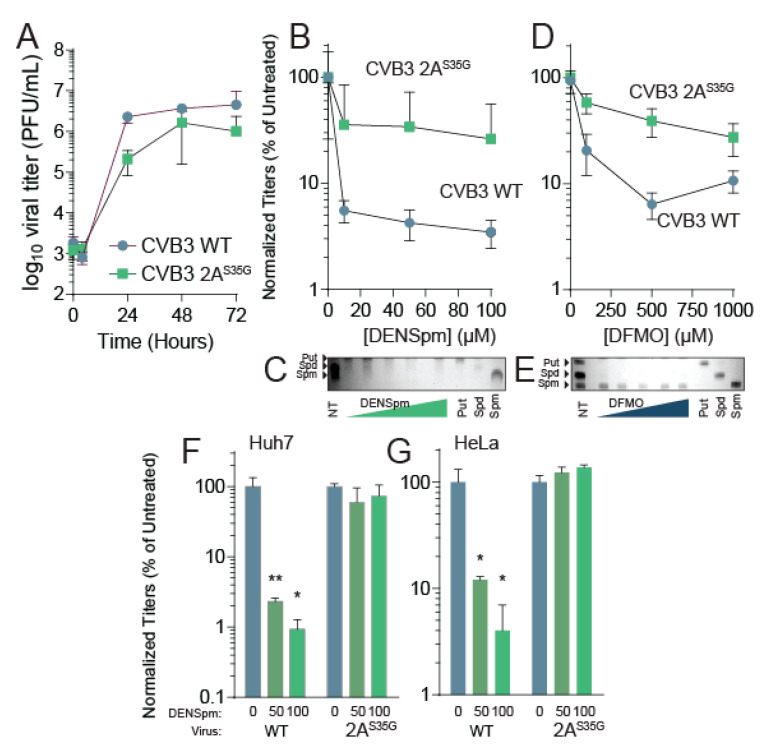
CVB3 2A^S35G^ confers resistance to DENSpm. (**A**) Vero cells were left untreated and were infected with CVB3 and 2A mutant protease at an MOI of 0.1. Samples were collected every 24 h and titered via plaque assay. (**B**) Vero cells were treated with increasing doses of DENSpm, from 10 μM to 100 μM, for 16 h prior to infection with wildtype CVB3 or 2A protease mutant. Viral titers were determined via plaque assay at 48 hpi. Viral titers were used to calculate the percent replication in DENSpm, by dividing the titer of the virus after infection of DENSpm-treated cells by the titer of the virus after infection of untreated cells at 48 hpi. (**C**) Thin layer chromatograms resolving the polyamines putrescine (Put), spermidine (Spm), and spermine (Spm) after treatment with DENSpm. Arrows indicate the band corresponding to each polyamine. (**D**) Vero cells were treated with increasing doses of DFMO, from 100 μM to 1 mM, for 4 days prior to infection with wildtype CVB3 or 2A protease mutant. Viral titers were determined via plaque assay at 48 hpi. Viral titers were used to calculate the percent replication in DFMO, by dividing the titer of the virus after infection of DFMO-treated cells by the titer of the virus after infection of untreated cells at 48 hpi. (**E**) Thin layer chromatograms resolving the polyamines putrescine (Put), spermidine (Spm), and spermine (Spm) after treatment with DFMO. (**F**) Huh7 cells and (**G**) HeLa cells were treated with increasing doses of DENSpm and infected with WT or 2A protease mutant as in (**B**). * *p* ≤ 0.05, ** *p* ≤ 0.01 using Student’s *t*-test (*n* ≥ 2), comparing treated samples to untreated controls. Error bars represent ± 1 SEM.

This 2A^S35G^ mutation was cloned in the CVB3 parental strain using mutagenic PCR on the CVB3 infectious clone. The presence of the mutation was verified, and infectious virus was made by transfecting the plasmid with the mutant into 293T cells expressing the T7 polymerase. After successfully recovering virus, we measured the replication kinetics of the mutant compared to the wildtype virus. To this end, untreated Vero cells were infected with either CVB3 WT or the 2A^S35G^ CVB3 at an MOI of 0.01. Supernatant was collected from the cells at different points of infection, and viral titers were determined (Figure 3A). The mutant virus showed similar replication kinetics to the wildtype virus although at slightly lower titers.

Because we isolated this mutant after passage in DENSpm, we anticipated that the 2A^S35G^ mutant may resist DENSpm-mediated viral restriction. To determine resistance to DENSpm, cells were treated with increasing doses of DENSpm 16 h prior to infection or left untreated. Cells were then infected with either wildtype or mutant CVB3 for 24 h. Supernatant was collected and titers were determined. Wildtype virus replication significantly reduced with DENSpm treatment compared to the untreated control; however, replication of the 2A^S35G^ mutant did not significantly reduce with DENSpm treatment compared to the untreated control suggesting the mutant gained resistance (Figure 3B). We confirmed that cellular polyamines were reduced with DENSpm treatment (Figure 3C).

Because DENSpm and DFMO both reduce cellular polyamine levels, we investigated whether the mutant was resistant to DFMO treatment as well. Vero cells were treated with increasing doses of DFMO 96 h prior to an infection or left untreated. The cells were then infected with either CVB3 wildtype or the mutant CVB3 for 24 h. The supernatant was collected, and viral titers were determined via plaque assay. As with DENspm, the replication of wildtype virus decreased significantly with DFMO treatment compared to control (Figure 3D). The 2A^S35G^ mutant virus did not have a significant decrease in replication with DFMO treatment compared to the untreated control, suggesting a partial resistance to DFMO as well. Again, we confirmed polyamine depletion by thin layer chromatography (Figure 3E). Thus, resistance to DENSpm via 2A^S35G^ confers resistance to DFMO-mediated polyamine depletion.

Vero cells lack an intact antiviral interferon (IFN) response, making them highly susceptible to viral infection [33]. Although these cells demonstrate both wildtype CVB3 sensitivity and 2A^S35G^ mutant resistance to DENSpm treatment, the impact of polyamine depletion on wildtype CVB3 in immunocompetent cells is yet to be determined. This immunocompetence may also have an impact on the 2A^S35G^ mutant’s ability to overcome this sensitivity as well. To address this, we performed additional dose responses with two cell lines that both contain intact IFN signaling, Huh7 and HeLa cells [34,35]. Huh7 and HeLa cells were treated with increasing concentrations of DENSpm 16 h pre-infection with either wildtype CVB3 or the 2A mutant at an MOI of 0.1. We found that in both Huh7 (Figure 3F) and HeLa (Figure 3G), wildtype virus titers were significantly reduced with DENSpm treatment; however, the 2A^S35G^ mutant was resistant, similar to our results in Vero cells.

### 3.4. CVB3 2A^S35G^ Exhibits Reduced Fitness Compared to Wildtype Virus In Vitro

**Figure 4:**
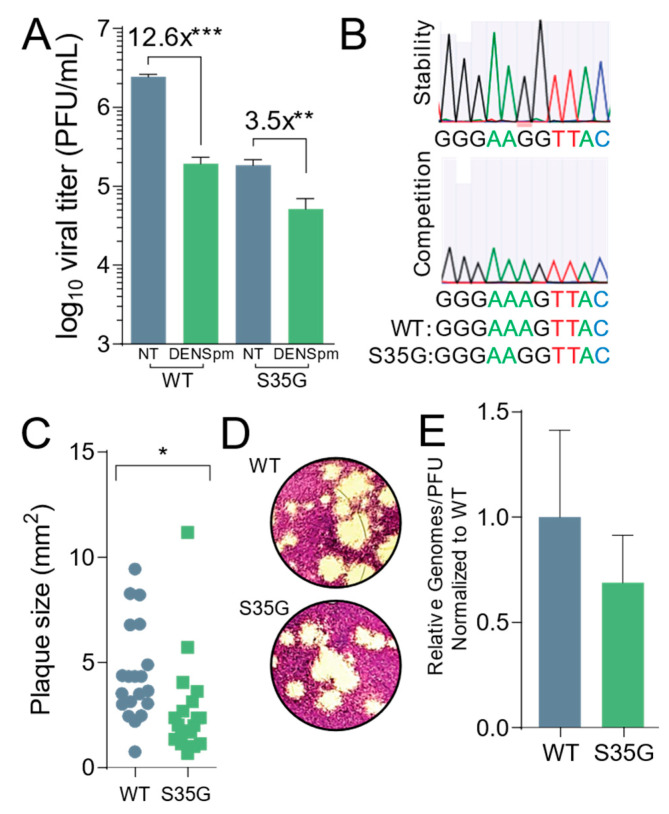
CBV3 2A^S35G^ exhibits reduced fitness in vitro. Veros cells were treated with 100 μM for 16 h or left untreated prior to being infected with wildtype and mutant CVB3 for 24 h, at which time (**A**) viral titers were determined via plaque assay. (**B**) WT and mutant CVB3 were used to infect untreated Vero cells for five passages at which time viral RNA was reverse transcribed, PCR amplified, and sequenced to measure stability and fitness of mutant. (**C**) Plaque sizes of virus mutants were determined after two-day plaque assay. (**D**) Pictures of plaques that were measured in (**C**). (**E**) Viral genomes in cell supernatant collected from untreated cells in (**A**) were determined by qPCR. The ratio of genomes-to-PFU was calculated by dividing the relative genomes by the titer in (**A**). * *p* ≤ 0.05, ** *p* ≤ 0.01, *** *p* ≤ 0.001 using Student’s *t*-test (*n* ≥ 3), comparing treated samples to untreated controls. Error bars represent ± 1 SEM.

Next, we wanted to compare the fitness and replication characteristics of the 2A^S35G^ mutant to wildtype virus. Passaging CVB3 in cells can potentially result in mutations with a greater or equal replication advantage, as previously observed [18,28]. To determine if this mutant had a change in fitness manifesting as a change in replicating virus, we measured titer and replication differences between the 2A^S35G^ mutant and wildtype virus. Vero cells were plated and treated with 100 μM DENSpm for 16 h or left untreated, and then infected with either the mutant or wildtype virus at an MOI of 0.1 for 24 h. The mutant virus exhibited lower titers compared to wildtype virus after a 24 h replication period (Figure 4A). However, DENSpm treatment had a smaller relative effect on viral replication for the mutant virus compared to the wildtype virus, with a 3.5-fold decrease in viral titers compared to 12.6-fold decrease for wildtype CVB3.

Importantly, we wanted to establish the stability of the mutant and consider its fitness. To ascertain the stability of the mutant, Vero cells were left untreated and infected with the CVB3 2A^S35G^ mutant at an MOI of 0.1. After 24 h, 1/10th of the supernatant from the infected cells was passaged into the next set of wells for 24 h. This was done for five passages, and the supernatant was collected. Viral RNA was extracted, purified, reverse transcribed, and sequenced. The samples were aligned with the parental genome, and we found that after multiple replication cycles the mutation remained and was stable (Figure 4B, above). We then performed a competition assay to look at the fitness of the mutant after multiple replication cycles. Vero cells were infected with an equal amount (PFU) of CVB3 wildtype and the protease mutant at an MOI of 0.1; 1/10th of the supernatant from the cells was passaged into the next set of cells for five passages. Viral RNA from the fifth passage was extracted, purified, reverse transcribed, and sequenced. The sequenced samples were aligned to the parental genome, and the wildtype CVB3 was the only virus present, suggesting that compared to the wildtype, the protease mutant had a reduced fitness (Figure 4B, lower).

Additionally, we measured the plaque sizes of both wildtype and mutant virus (Figure 4C,D). We observed a small yet significant decrease in plaque size of the protease mutant, suggesting that this mutant has a decreased fitness. To establish if the protease mutant produced the same amount of infectious particles, the ratio of viral genomes to infectious particles was measured (Figure 4E). Cells were left untreated and infected with wildtype or protease mutant virus at an MOI of 0.1. Viral titers were determined by plaque assay, and viral RNA was extracted, purified from the supernatant, and reverse transcribed. The amount of viral genomes was quantified by qPCR, and the ratio of genomes to infectious virus (PFU) was measured. No significant difference was found between the wildtype and protease mutant.

### 3.5. DENSpm Reduces 2A Protease Activity

**Figure 5:**
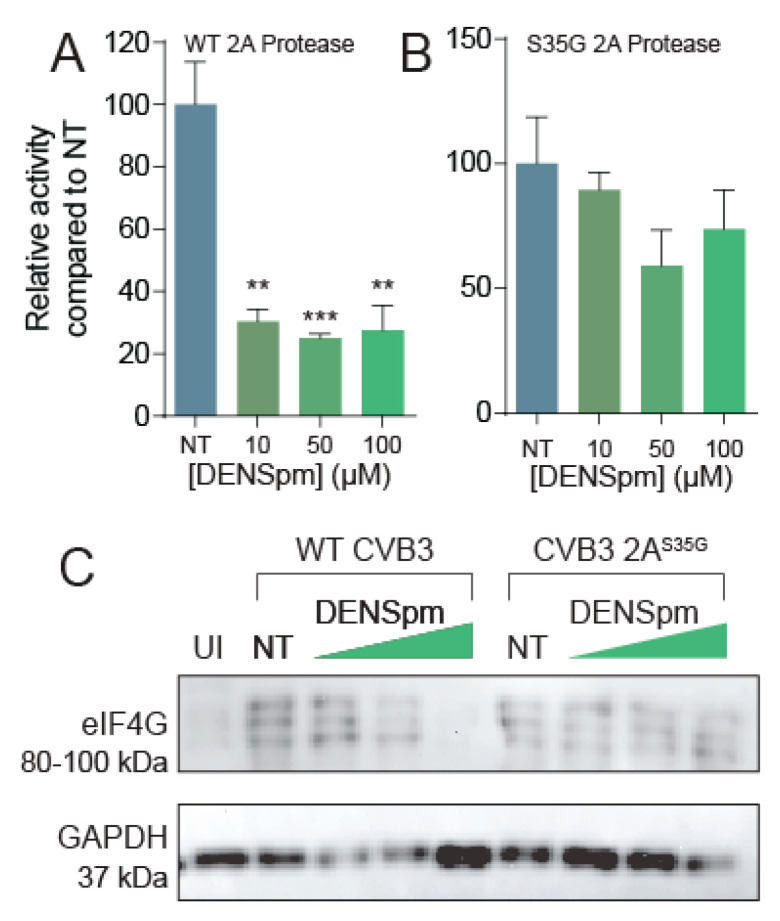
DENSpm reduces 2A protease activity. Split luciferase protease activity reporter systems were cloned and co-transfected with (**A**) wildtype 2A or (**B**) 2A^S35G^ mutant into Vero cells left untreated or treated with increasing doses of DENSpm. Firefly luciferase activity was measured 24 h later and normalized to renilla luciferase transfection efficiency control and subsequently normalized to untreated cell transfection. (**C**) Vero cells were left untreated or treated with increasing doses of DENSpm for 16 h prior to infection with wildtype CVB3. Total cellular protein was collected 24 hpi and analyzed via Western blot for eIF4G and β-actin. ** *p* ≤ 0.01, *** *p* ≤ 0.001 using Student’s *t*-test (*n* = 2), comparing treated samples to untreated controls. Error bars represent ± 1 SEM.

Prior work demonstrated that polyamines, specifically spermidine, enhances chymotrypsin activity [36]; however, whether polyamines affect enterovirus proteases, like 2A and 3C of CVB3, has only recently been explored [18]. To determine if polyamines are necessary for protease activity, we treated Vero cells with varying concentrations of DENSpm for 16 h or left them untreated. We then used a dual luciferase reporter system previously described [18] to measure 2A protease activity without polyamines present (Figure 5A,B). We observed a significant decrease in protease activity at all concentrations of DENSpm; thus, CVB3 wildtype 2A protease is dependent on polyamines for function.

Because this 2A^S35G^ protease mutation confers resistance to polyamine depletion, and we have seen altered 2A protease activity in mutants previously [18], we hypothesized that the 2A^S35G^ mutant has modulated protease activity to overcome CVB3’s sensitivity to polyamine depletion. To investigate this hypothesis, we used our luciferase protease assay and cloned the 2A^S35G^ mutant protease into a pCMV expression plasmid. Firefly luciferase activity was measured 24 h after transfection and normalized to a transfection control (Renilla luciferase). We found that 2A^S35G^ protease activity did not significantly decrease with DENSpm treatment (Figure 5B), suggesting that this mutant confers protease resistance to polyamine depletion.

To determine if the 2A^S35G^ mutant protease has enhanced proteolytic cleavage of cellular targets during infection, cells were treated with DENSpm 16 h prior to infection or left untreated. Cells were then infected with wildtype or protease mutant CVB3 at an MOI of 5 for 24 h. Cellular lysates were collected, and eIF4G cleavage was analyzed via Western blot (Figure 5C). We observed the appearance of three bands at approximately 95 kDa, corresponding to cleaved eIF4G, specifically during viral infection, not present in uninfected (UI) sample. This eIF4G signal was decreased with increasing DENSpm concentration with wildtype CVB3 infection. In contrast, cleaved eIF4G was observed uniformly with mutant CVB3 infection, suggesting that the 2A^S35G^ mutation maintains the ability to cleave eIF4G despite DENSpm-mediated polyamine depletion.

## 4. Discussion

**Figure 6:**
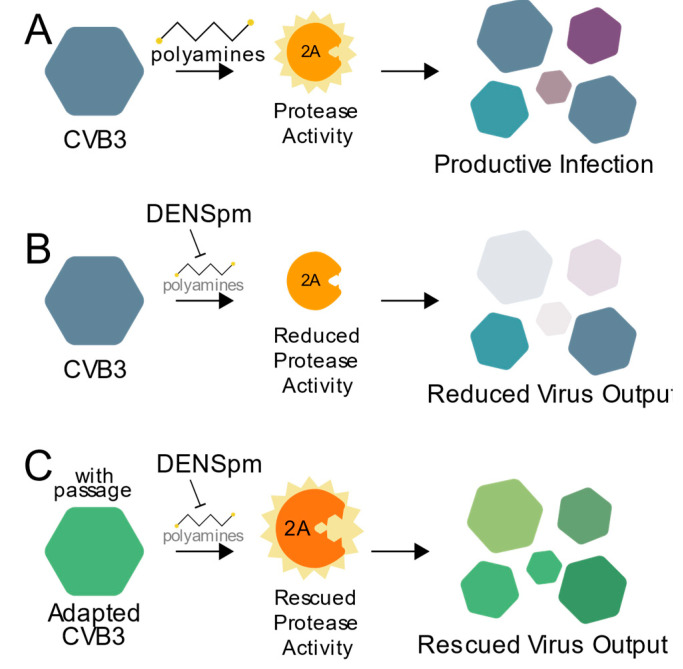
Working model. (**A**) CVB3 relies on polyamines for efficient 2A protease activity to promote viral replication. In the presence of an inhibitor of polyamines, like DENSpm (**B**), protease activity and viral replication are reduced. (**C**) CVB3 overcomes polyamine depletion over successive rounds of replication via mutation of the 2A protease to facilitate rescued virus replication.

Polyamines have diverse and important roles during infection, and here we highlight a critical role for polyamines in protease activity during enterovirus infection. As summarized in Figure 6, CVB3 protease 2A activity relies on cellular polyamines (Figure 6A). Thus, when polyamines are depleted, through compounds like DENSpm or DFMO, protease activity is inhibited, and viral replication is reduced (Figure 6B). However, escape mutants isolated after replicating CVB3 in cells depleted of their polyamines, like 2A^S35G^, overcome this sensitivity to polyamine depletion allowing for protease activity along with subsequent viral replication (Figure 6C). Our data not only show CVB3 and viral proteins’ reliance on cellular molecules for infection, but also the potential risk of antiviral resistance.

Targeting proteases to combat viral infection shows significant promise. Recent studies have shown that targeting CVB3 proteases are a potential effective antiviral therapy and significantly decrease viral replication, though they are not used clinically [27,28]. Protease inhibitors have not only been effective antiviral therapies for CVB3 but also for other viruses, including HIV, hepatitis C virus, and noroviruses [37,38]. Our data suggest that polyamines contribute to protease activity in vitro during enterovirus infection, and this highlights an opportunity for targeting protease activity indirectly, by targeting host polyamines. Because there are no antiviral therapies currently available for CVB3 infection, targeting CVB3 proteases, potentially through polyamine depletion, may hold significant promise.

Drug resistance and viral escape from antiviral therapy is a rising threat to health and patient care. The 2A^S35^ escape mutant we identified in this study provides insight into both viral evolution and how potential antivirals (in this case, DENSpm) work during infection. However, they also show how quickly a virus gains resistance to treatment. Others have described the genetic pliability of picornaviruses, like rhinoviruses, and the subsequent ability to overcome antiviral treatments [39]. Depending on the stability and fitness of the escape mutants, antiviral resistance can be devastating to a patient where not only is the virus resistant to treatment, but it is also stable after multiple replication cycles similar to this 2A^S35G^ escape mutant has shown to be in vitro. However, further studies need to be performed to determine if these results can be translated in vivo and this mutant and other similar mutants maintain this resistance and stability. Further, additional studies in cells pertinent to CVB3 replication, such as cardiac cells, will be necessary to test the function of polyamine inhibitors in more physiologically relevant cells.

We previously showed that other escape mutants in the CVB3 proteases exhibit stability and no loss in fitness compared to wildtype virus [18]. In contrast, the mutant described here, 2A^S35G^, exhibits reduced fitness, suggesting that the serine at position 35 is important for viral fitness that may be sacrificed to promote replication in polyamine-depleted cells. Because resistance to polyamine-depleting drugs is possible, combination therapy may be an effective solution against these infections, and our work highlights the need for combination therapy (or other mitigation techniques) to prevent the emergence of the mutants we have identified.

Polyamines show significant promise as an antiviral target, and this and several other studies show the importance of polyamines in viral infections [7,8]. Repurposing drugs is an efficient way to accelerate antiviral development, as drugs with extensive characterization are better understood in terms of their pharmacokinetics and toxicity profile. DENSpm has undergone clinical trials, and DFMO is FDA approved. DFMO has already been shown to be effective at limiting viral titers in vitro and in vivo [10,17,18], and DENSpm is well tolerated in patients, though not yet approved [40]. Important questions remain concerning the viability of polyamine depletion as an antiviral therapy, including drug dosage and delivery mechanisms. These studies further highlight that even if polyamine depletion is a viable antiviral strategy, viruses mutate to overcome treatment, and additional precautions would be necessary in successfully treating infected patients, mitigating the emergence of resistant mutants.

## Data Availability

Not applicable.
